# Results of the Surgical Approach of Idiopathic Scoliosis in Adolescents and Postoperative Quality of Life: Systematic Literature Review

**DOI:** 10.1055/s-0044-1785658

**Published:** 2024-06-22

**Authors:** Bianca Gabriella de Oliveira, Thiago Silva Moura, Guilherme de Brito Lira Dal Monte, Bruno dos Santos Souza, Leonardo da Costa Borduchi, Laís Cristina Pereira da Silva

**Affiliations:** 1 Universidade Salvador (UNIFACS), Salvador, BA, Brasil; 2 Ortopedia e Traumatologia, Centro Universitário UNIFACISA, Campina Grande, PB, Brasil; 3 Ortopedia e Traumatologia, Hospital Geral Clériston Andrade, Feira de Santana, BA, Brasil; 4 Ortopedia e Traumatologia, Centro Universitário Padre Albino (UNIFIPA), Catanduva, SP, Brasil

**Keywords:** adolescent, orthopedic surgery, scoliosis

## Abstract

Adolescent idiopathic scoliosis is considered the most severe and common spinal deformity, affecting children and adolescents still in the neuropsychomotor development phase before they reach skeletal maturity.

This study aimed to evaluate the surgical approach to adolescent idiopathic scoliosis (AIS), considering the results associated with the reduction of pathological curvature, pulmonary function, and repercussions on the quality of life of adolescents undergoing such treatment.

Systematic literature review, with a quantitative and qualitative approach to the data collected, structured according to the guidelines of the Preferred Reporting Items for Systematic Reviews and Meta-Analyses (PRISMA), carried out in the databases linked to the Medical Literature Analysis and Retrieval System Online (MEDLINE) and Latin American and Caribbean Health Sciences Literature (LILACS). The total sample of the studies was 638 adolescents with AIS, with a mean age of 14.93 years ± 1.24. The mean correction of the main pathological curvature in the studies was 55.06% ± 12.24.

In all of the selected studies using posterior spinal fusion to correct AIS, there was a significant reduction in pathological curvatures (> 49%), and the recurrence of curvature in none of the studies exceeded a pathological gain of more than 5%. As for lung function, the studies showed significant increases in forced expiratory volume in 1 second (FEV1) and forced vital capacity (FVC) in patients with severe AIS, and no pulmonary function losses were reported after surgery to correct AIS.

## Introduction

Adolescent idiopathic scoliosis (AIS) is considered the most severe and common spinal deformity that affects children and adolescents during the neuropsychomotor development phase before they reach skeletal maturity, causing significant alterations in the sagittal curvatures of the spine. 1

This is a three-dimensional spine deformity in which there is a process of lateral inclination and axial rotation of the vertebral bodies at an angle of more than 10 degrees, measured according to the Cobb method. 2 To measure the Cobb angle (CA), two-dimensional or three-dimensional X-rays (X/2D or 3D) are taken in the sagittal and anteroposterior profiles. The CA is then calculated from perpendicular lines from the projection of a tangent line at the top of the vertebra, indicating the beginning of the curvature of the spine, and another tangent line at the base of the vertebra, indicating the end of the curvature of the spine. 3

Various studies indicate that the prevalence of AIS varies between 0.35 and 5.2%. 4 5 In a study conducted in São Paulo, Brazil, the overall prevalence of AIS in adolescents was 1.5%, considering a total sample of 2,562 adolescents aged between 10 and 14. There was also a higher prevalence in females (2.2%) than males (0.5%). 6

Curvatures usually progress in up to ⅔of patients before they reach skeletal maturity, and angles greater than 50° are associated with various pathophysiological repercussions. 7 The literature reports high levels of disability, intense pain, and significant cardiovascular impairment. 8 However, recent data shows that idiopathic scoliosis has significant variability in the type of curvature, location, and etiopathogenesis, which can lead to different clinical outcomes and functional limitations. 9

The risk factors for progression of the curvature include age under 12, premenarcheal girls with CA curves ≥ 20°, as well as the presence of double and thoracic curves, and Risser grade 0 or 1. 10

The approach to AIS cases aims to reduce or stop the progression of the curvature during puberty and prevent cardiorespiratory dysfunctions and vertebral pain syndromes. Among the available alternatives are orthotics, therapeutic exercise protocols, and a surgical approach. Surgical correction is indicated in patients with skeletal immaturity and curves with CA ≥ 50°, although some individual peculiarities may lead to a surgical approach in patients with CA between 40 and 50° or even individuals who already have skeletal maturity and CA ≥ 50°. 11 12

The surgical approach to AIS aims to prevent the progression of the curvature and achieve permanent correction of the deformity. The surgical treatment aims to maintain the spine's stability in the sagittal and coronal planes, preserving as many mobile segments as possible. 13 14 The present study aimed to evaluate the surgical approach to AIS, considering the results associated with curvature reduction, pulmonary function, and repercussions on the quality of life of adolescents undergoing this treatment.

## Materials and Methods

A systematic literature review, with a quantitative and qualitative approach to the data collected, was structured according to the Preferred Reporting Items for Systematic Reviews and Meta-Analyses (PRISMA) 12 guidelines. A checklist was then drawn up to analyze the results. A four-stage flow diagram was used to analyze the data in detail.

The search for studies that met the established criteria took place in March 2023 in databases linked to the *Medical Literature Analysis and Retrieval System Online* (MEDLINE) and Latin American and Caribbean Literature in Health Sciences (LILACS), using the SPICE strategy 13 to identify the relevant studies:

- *Setting* : Patients with AIS
- *Perspective* : Individuals with arthrogryposis and clubfoot;
- *Intervention* : Surgical correction;
- *Comparison* : Reduction in pathological curvature, improvement in pain, improvement in quality of life and lung function;
- *Evaluation* : Rate or occurrence of pathological curvature recurrence or lung function worsening.

The medical subject heading MeSH terms were used in combination, according to the following structure:

- *Scoliosis* AND *Adolescent* AND *Orthopedic* Procedures

Subsequently, the studies were sorted according to their subject matter, restricting them to studies that dealt with the surgical correction of adolescent idiopathic scoliosis.

### Inclusion and Exclusion Criteria

Studies that met the following criteria were included: (1) studies with humans, age group < 18 years; (2) patients diagnosed with AIS; (3) studies addressing patients treated with surgical correction; (4) studies published between 2018 and 2023, and (5) original studies, preferably randomized ones.

Studies with the following criteria were excluded: (1) experimental studies using animal models; (2) non-original studies - literature reviews; (3) opinion studies; (4) studies that addressed other approaches to managing AIS without a surgical approach; (5) studies published more than 5 years ago, and (6) studies that did not meet the other inclusion criteria mentioned above.

The studies were searched and selected by two reviewers who independently analyzed them. Initially, using the MeSH terms mentioned above and Boolean operators, studies published in the last 5 years (2018-2023) were selected, followed by an analysis of titles and abstracts. This stage excluded studies using animal models, opinion articles, studies not considering the surgical approach to AIS, and literature reviews.

Once this stage had been completed, the full texts of the articles were retrieved for analysis of the other inclusion and exclusion criteria. Duplicate citations and studies not corresponding to the proposed review parameters were also excluded. Possible disagreements were resolved by discussion with a third reviewer, and inclusion was decided after consensus with the two primary reviewers.

Epidemiological and demographic data were extracted using a Microsoft Excel (Microsoft Corp., Redmond, WA, USA) spreadsheet, including parameters such as the number of patients, the initial and final degree of curvature of the AIS, treatment strategies, recurrences, complications, and the results obtained.

The *systematic* review protocol *was registered in the* International Prospective Register of Systematic Reviews (PROSPERO) under the number ID CRD42023429455 to improve the quality and suitability of the results to the proposed objectives.

## Results

**Fig. 1:**
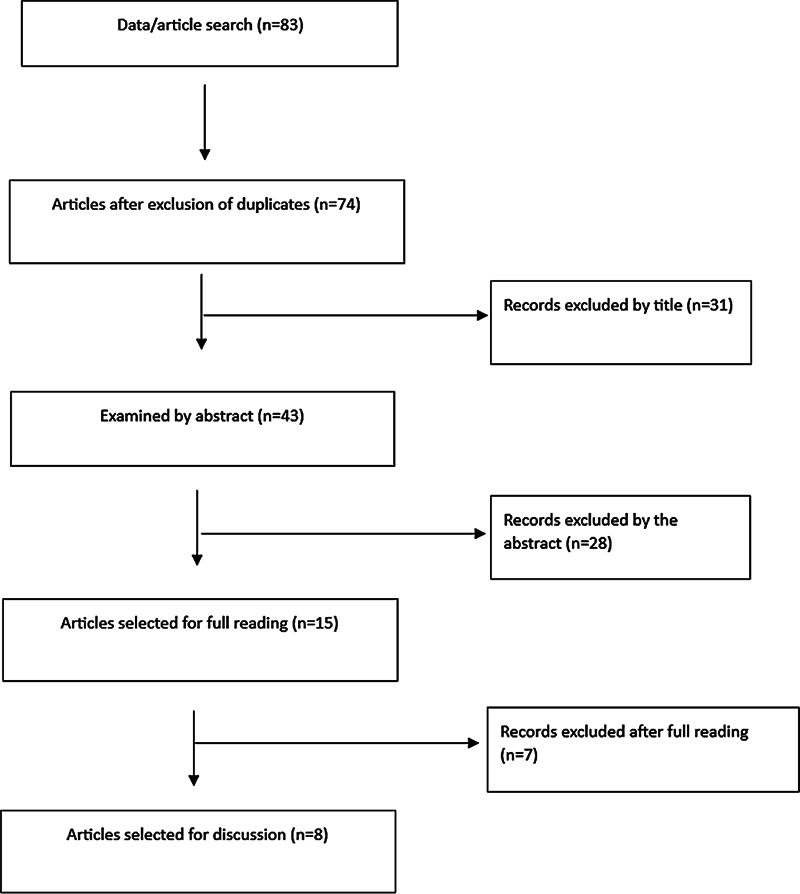
Screening and selection of studies according to the Preferred Reporting Items for Systematic Reviews and Meta-Analyses (PRISMA) methodology. **Source:** Elaborated by the authors (2023).

After screening according to the PRISMA protocol, 83 studies were initially retrieved, of which 8 were clinical practice guidelines, 7 were systematic reviews, and 21 were opinion studies or narrative reviews. Off-topic studies ( *n* = 13) and studies of a conservative approach to AIS ( *n* = 25) were also excluded. The following variables were considered for the analysis of 8 studies: techniques used, curvature reduction, improvement or maintenance of lung function, pain reduction or improvement in quality of life ( Fig. 1 ).

The total sample of these studies was 638 adolescents with AIS, with a mean age of 14.93 years ± 1.24. The average correction of the main pathological curvature in the studies was 55.06% ± 12.24 ( Table 1 ). 15 16 17 18 19 20 21

**Table 1:** Correlation between age at surgery and mean correction of the main pathological curvature in adolescents with adolescent idiopathic scoliosis

Study	Sample	Average age	Mean correction of pathological curvature
** Byun et al. 15**	35 patients with thoracic AIS	14.9 years	49.19%
** Grabala et al. 18**	195 adolescents with AIS	14.3 years	59%
** Zhang et al. 19**	11 adolescents with CA > 130°	13.36 years	40.39%
** Altaf et al. 25**	37 adolescents with AIS	14.6 years	33.4%
** Santos et al. 16**	41 adolescents with AIS	17.8 years	68%
** Sapriza et al. 17**	19 patients with AIS	14 years old	60%
** Garcia et al. 20**	278 AIS patients operated on with selective, traditional, and multiple fixations.	15 years	71%
** Dittmar-Johnson et al. 21**	22 patients underwent corrective surgery for AIS	15.5 years	59.5%

Abbreviations: AIS, adolescent idiopathic scoliosis; CA, Cobb's angle.

Source: Elaborated by the authors (2023).

**Fig. 2:**
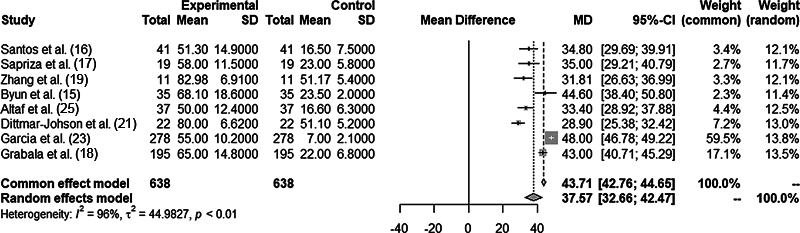
Mean correction of pathological curvature using the Cobb angle pre-and postoperatively.

Fig. 2 shows the analysis of the mean correction of the pathological curvature using the pre and postoperative CAs. 15 16 17 18 19 20 21

## Discussion

### Average correction of pathological curvatures

The main objectives of the surgical approach to AIS are to correct the pathological curvatures coronally and sagittally and to prevent their progression. Thus, it is expected that after treatment, there will be correction of the main primary curve and secondary curves while maintaining adequate lumbar lordosis and thoracic kyphosis. The treatment aims to balance fusing as few mobile segments as possible and reducing/correcting the existing deformity. In addition to correcting the deformities, the expected results are to improve and maintain lung function, reduce pain, and improve quality of life. 14

All the selected studies used posterior spinal fusion techniques for AIS correction. One of the techniques described is posterior spinal fusion with segmental pedicle screw in a lumbar lesion and segmental hook and wiring in a thoracic lesion (hybrid construction) using the ISOLA instrumentation system (DePuy AcroMed, Raynham, MA, USA) without thoracoplasty. 15 Direct vertebral rotation (DVR) techniques associated with type-1 osteotomy have also been reported, 16 Thoracic arthrodesis, thoracolumbar arthrodesis, and lumbar arthrodesis. 17 22 23

### Lung Function after Surgery

Posterior and anterior spinal fusion techniques can correct AIS. Although both types of approach offer correction of pathological curvatures, some studies suggest that the anterior approach with thoracotomy may result in a more significant impairment of lung function. Pulmonary function in the studies was assessed according to the values of forced vital capacity (FVC) and forced expiratory volume in 1 second (FEV1). 15 24

In a 15-year longitudinal study carried out by Byun et al. 15 among patients who did not have preoperative FVC impairment ( *n* = 35), no statistically significant differences were found in the mean preoperative (74.0 ± 19.8%) and postoperative (76.4 ± 16.0%) percentages, *p* = 0.63. On the other hand, patients who already had preoperative impairment benefited from the surgical approach. 15

In a similar approach, including 39 patients with AIS aged between 10 and 21 years, it was observed that after the restoration of thoracic kyphosis, there was an improvement in FEV1 from 2.74 to 2.98L ( *p* = 0.005) and FVC from 3.23 to 3.47L ( *p* = 0.008). However, total lung capacity did not change after 5 years of follow-up. 24

In one of the studies, including 88 patients with severe AIS and 107 with moderate AIS and an average deformity correction of 59%, significant gains in lung function were reported. After 2 years, the severe AIS group observed an improvement in FVC of 69.9% and FEV1 of 81%. In the moderate AIS group, FEV1 improved significantly (76.9%) after 2 years of follow-up. 19

Concerning the type of vertebral fixation, one of the studies discussed different possibilities for vertebral fixation, considering the degree of AIS correction required. The study refers to treating three groups of patients with AIS who underwent three different fixation approaches: traditional, selective, and multiple. In the first group operated on, the entire length of the structured curvatures was fixed, known as traditional fixation; in the selective fixation group, only the main curve was fixed to preserve vertebral mobility. In the third group, multiple fixation occurred according to the flexibility test, defining the degree of correction required and the vertebrae to be fixed. In the study, the traditional and multiple fixation techniques corrected 75% and 78% of the pathological curvature, respectively, while selective fixation corrected 60%. Considering all the cases, the study showed an average correction of 71% ± 7.87 of the pathological curvatures of the AIS. The study did not address the quality of life or pulmonary function associated with the results obtained. 20

Sapriza et al. 17 followed 19 patients with AIS who underwent surgical correction by posterior spinal fusion over 9.5 years, with an average of 10 fused intervertebral spaces per patient. The mean preoperative angle was 58 (range 90–42). The mean postoperative angle value before the end of the 1 ^st^ year was 23, for a correction rate of 60%. The average value of the angle at the end of the follow-up was 26. Therefore, an average correction of 5% was lost at the end of the follow-up. Only 3 cases had a loss of more than 10%. The adolescents considered their quality of life and body appearance improved significantly after the follow-up period. The authors did not analyze pulmonary repercussions in the study.

Santos et al. 16 conducted a cross-sectional observational study with 43 adolescents (mean age 17.8 years) with a CA between 35 and 55° (mean CA 51.5° ± 13.7°). A posterior approach was used with classic midline access. After subperiosteal dissection of the muscles, a Schwab type-1 osteotomy was performed. In addition, the most proximal level of arthrodesis and Schwab type-2 osteotomies were performed on the periapical vertebrae according to the subjective assessment of curvature reducibility during the procedure. A 68% correction of the primary thoracic curve was obtained. The study did not report quality of life, pain, or lung function data.

In a study of 195 adolescents with AIS, 88 of whom were classified as severe (AC > 130°) and 107 as moderate AIS, who underwent a posterior approach for surgical correction of the AIS, a mean correction of the deformity after the surgical approach of 59% and a reduction in the number of individuals with pain complaints from 36 to 8% were observed. In patients with severe AIS ( *n* = 88), the average curvature was reduced from 131 to 61° and the average thoracic kyphosis from 83 to 35°. Among patients with moderate AIS, the reduction in mean curvature was 60 to 18°, and there was no reduction in thoracic kyphosis in this group. 18

In a study looking at posterior-only surgical correction with halo-femoral traction, 11 adolescents with AC > 130° were evaluated over a mean follow-up period of 32.18 ± 8.17 months. The mean preoperative coronal CA of the greater curve was 139.01° ± 5.83°, and the mean flexibility was 17.21% ± 3.33%. After the approach, the coronal AC of the greater curvature was reduced to 82.98° ± 6.91°, with a correction rate of 40.39%. At the final follow-up, the rate of corrective loss of CA was only 0.72%. 19

In a study of 37 patients with moderate AIS (AC between 50 and 129°) who underwent thoracoplasty, a mean preoperative curvature of 50.0° ± 12.4° was observed, with a subsequent reduction to 16.6° ± 6.3°. The mean correction of deformity was 33.4%, with a mean improvement in FVC of 55.4% and FEV1 of 72%. In the study, the authors used the Scoliosis Research Society (SRS) 22 score to analyze the adolescents' quality of life and found an improvement in the score from 3.8 to 4.3 after surgery. 25

A study by Dittmar-Johnson et al. 21 included 22 patients undergoing posterior spinal fusion for AIS correction with pathological curves greater than 45°. The average age of the patients was 15.5 years. The SRS 22 questionnaire generated the following mean scores: pain 4.6, function 4.3, self-image 4.41, mental health 4.89, and satisfaction 5.0. Therefore, the surgical intervention was considered to have led to a good quality of life in the five parameters assessed.

The SRS 22 had been used previously in a longitudinal study with a 10-year follow-up of 109 adolescents with AIS treated with a surgical approach. When all patients were included, the mean preoperative CA of the major curves in the frontal plane was 60.8° +/−17.5°. Major curves corrected by 38.7° +/−22.1% on flexion radiographs, postoperatively achieved a correction of 64.0° +/−15.8%. At the last follow-up visit, 10.3° +/−10.8° of loss of correction were recorded on the principal curves in the frontal plane with 50.5° +/−23.1% final correction rate. In addition, the mean postoperative and final kyphosis angles and lumbar lordosis angles were 37.7° +/−7.4°, 37.0° +/−8.4°, 37.5° +/−8.7°, and 36.3° +/−8.5°, respectively. Overall, 4 patients (3.7%) experienced implant failure. Early superficial infection was observed in 3 patients (2.8%). Overall, the mean SRS-22 questionnaire scores for general self-image, function, mental state, pain, and satisfaction with treatment were 3.8 +/−0.7, 3.6 +/−0.7, 4.0 +/−0.8, 3.6 +/−0.8, and 4. 6 +/−0.3, respectively, at the last follow-up visit. Given the results, the authors consider that the surgical approach efficiently corrects deformities in the frontal and sagittal planes and trunk balance, resulting in a better quality of life. 26

## Conclusions

In all the selected studies using posterior spinal fusion to correct AIS, there was a significant reduction in pathological curvatures (> 49%), and in none of the studies did the recurrence of curvature exceeded a pathological gain of more than 5%. As for pulmonary function, the studies showed significant increases in FEV1 and FVC in patients with severe AIS, and no pulmonary function losses were reported after surgery to correct AIS. Finally, most studies reported improved quality of life and reduced pain after surgery.
