# Supplementary material for: Results of the Surgical Approach of Idiopathic Scoliosis in Adolescents and Postoperative Quality of Life: Systematic Literature Review
Source: Rev Bras Ortop (Sao Paulo). 2024 Jun 22;59(3):e358–63. doi: 10.1055/s-0044-1785658 (PMC11193578; doi:10.1055/s-0044-1785658)
Supplement: Related texts

# Research article: Resultados da abordagem cirúrgica da escoliose idiopática em adolescentes e qualidade de vida pós-operatória: Revisão sistemática de literatura

**Keywords:** adolescente, cirurgia ortopédica, escoliose

## Abstract

A escoliose idiopática do adolescente (EIA) é considerada a deformidade vertebral de maior gravidade e ocorrência, que acomete crianças e adolescentes ainda na fase de desenvolvimento neuropsicomotor, antes de atingirem a maturidade esquelética.

Este estudo teve como objetivo avaliar a abordagem cirúrgica da EIA, considerando resultados associados à redução de curvatura patológica, função pulmonar e repercussões na qualidade de vida dos adolescentes submetidos a tal tratamento.

Revisão Sistemática de literatura, com abordagem quanti-qualitativa dos dados coletados, cuja estruturação se deu conforme as orientações de *Preferred Reporting Items for Systematic Reviews and Meta-Analyses* (PRISMA) realizada nas bases de dados vinculadas à *Medical Literature Analysis and Retrieval System Online* (MEDLINE) e Literatura Latino-Americana e do Caribe em Ciências da Saúde (LILACS). A amostra total dos estudos foi de 638 adolescentes com EIA, com uma idade média de 14,93 anos ± 1,24.

A correção média da curvatura patológica principal nos estudos foi de 55,06% ± 12,24. Em todos os estudos selecionados com fusão espinhal posterior para correção da EIA verificou-se redução significativa das curvaturas patológicas (> 49%), sendo que a reincidência de curvatura em nenhum dos estudos superou um ganho patológico superior a 5%. Quanto à função pulmonar, os estudos apontaram ganhos significativos de volume expiratório forçado em 1 segundo (VEF1) e capacidade vital forçada (CVF) em pacientes com EIA grave. Além disso, não foram relatados prejuízos de função pulmonar após intervenção cirúrgica para correção da EIA.

## Introdução

A escoliose idiopática do adolescente (EIA) é considerada a deformidade vertebral de maior gravidade e ocorrência, que acomete crianças e adolescentes ainda na fase de desenvolvimento neuropsicomotor, antes de atingirem a maturidade esquelética, promovendo importante alteração das curvaturas sagitais da coluna vertebral. 1

Trata-se de uma deformidade tridimensional da coluna vertebral, em que há um processo de inclinação lateral e rotação axial de corpos vertebrais em angulação superior a 10 graus, medido segundo método de Cobb. 2 Para medir o ângulo de Cobb (AC) são realizadas radiografias bidimensionais e/ou tridimensionais (raio X/2D ou 3D) nos perfis sagital e anteroposterior. Calcula-se então o aAC a partir de linhas perpendiculares da projeção de uma linha tangente no topo da vértebra, indicando o início da curvatura da coluna, e de outra linha tangente na base da vértebra, que indica o fim da curvatura da coluna vertebral. 3

Diversos estudos apontam que a prevalência da EIA varia entre 0,35 e 5,2%. 4 5 Em estudo realizado no estado de São Paulo, Brasil, a prevalência geral de EIA em adolescentes foi de 1,5%, considerando uma amostra total de 2.562 adolescentes entre 10 e 14 anos. Observou-se ainda maior prevalência em mulheres (2,2%) do que em indivíduos do sexo masculino (0,5%). 6

As curvaturas costumam progredir em até ⅔dos pacientes antes de atingirem a maturidade esquelética, sendo que ângulos superiores à 50° estão associados à diversas repercussões fisiopatológicas. 7 São relatados na literatura elevada incapacidade, quadros álgicos intensos, além de importante comprometimento cardiovascular. 8 Entretanto, dados recentes apontam que a escoliose idiopática possui grande variabilidade quanto ao tipo de curvatura, localização e etiopatogenia, que pode determinar diferentes desfechos clínicos e limitações funcionais. 9

Dentre os fatores de risco para progressão da curvatura destacam-se idade inferior a 12 anos, meninas em pré-menarca com curvas do AC ≥ 20°, bem como a presença de curvas duplas e torácicas, além de grau 0 ou 1 de Risser. 10

A abordagem dos casos de EIA busca reduzir ou parar a progressão da curvatura durante a puberdade, além de prevenir disfunções cardiorrespiratórias e síndromes dolorosas vertebrais. Dentre as alternativas disponíveis atualmente destacam-se a ortotetização, protocolos de exercícios terapêuticos e abordagem cirúrgica. Indica-se a correção cirúrgica em pacientes com imaturidade esquelética e curvas com AC ≥ 50°, embora algumas peculiaridades individuais podem levar à abordagem cirúrgica em pacientes com AC entre 40 e 50° ou ainda, indivíduos que já apresentem maturidade esquelética e AC ≥ 50°. 11 12

A abordagem cirúrgica da EIA busca impedir a progressão da curvatura e atingir uma correção permanente da deformidade. É sempre objetivo do tratamento cirúrgico a manutenção da estabilidade da coluna nos planos sagital e coronal, preservando o máximo possível de segmentos móveis. 13 14 O presente estudo teve como objetivo avaliar a abordagem cirúrgica da EIA, considerando resultados associados à redução de curvatura, função pulmonar e repercussões na qualidade de vida dos adolescentes submetidos a tal tratamento.

## Materiais e Métodos

Revisão sistemática de literatura, com abordagem quanti-qualitativa dos dados coletados, cuja estruturação se deu conforme as diretrizes do *Preferred Reporting Items for Systematic Reviews and Meta-Analyses* (PRISMA), 12 sendo posteriormente estruturada uma lista de verificação para análise dos resultados. Para análise pormenorizada dos dados utilizou-se um diagrama de fluxo de quatro etapas.

A busca por estudos que contemplassem os critérios estabelecidos se deu em março/2023 em bases de dados vinculadas à *Medical Literature Analysis and Retrieval System Online* (MEDLINE) e à Literatura Latino-Americana e do Caribe em Ciências da Saúde (LILACS) sendo utilizada a estratégia SPICE 13 para identificação dos estudos relevantes:

- *Setting* (cenário): Pacientes com EIA;
- *Perspective* (perspectiva): Indivíduos com artrogripose e pé torto equinovaro;
- *Intervention* (intervenção): Correção cirúrgica;
- *Comparison* (comparação): Redução da curvatura patológica, melhora da dor, melhora da qualidade de vida e função pulmonar;
- *Evaluation* (avaliação): Taxa ou ocorrência de recorrência da curvatura patológica e/ou piora da função pulmonar.

Os descritores em ciências da saúde (DECS) foram utilizados de forma combinada, conforme a seguinte estrutura:

- *Escoliose* ( *Scoliosis* ) E *Adolescente* ( *Adolescent* ) E *Cirurgia* *Ortopédica* ( *Orthopedic Procedures* )

Posteriormente, os estudos foram triados conforme temática abordada restringindo-se aos estudos que versavam sobre a correção cirúrgica da EIA.

## Critérios de Inclusão e Exclusão

Foram incluídos estudos que atendiam aos seguintes critérios: (1) estudos com humanos, grupo etário < 18 anos; (2) pacientes com diagnóstico de EIA; (3) estudos abordando pacientes tratados com correção cirúrgica; (4) estudos publicados entre 2018 e 2023, e (5) estudos originais, preferencialmente randomizados.

Foram excluídos estudos com os seguintes critérios: (1) estudos experimentais com modelos animais; (2) estudos não originais – revisão de literatura; (3) estudos de opinião; (4) estudos que abordavam outras condutas para manejo da EIA sem abordagem cirúrgica; (5) estudos publicados há mais de 5 anos, e (6) estudos que não atendiam aos demais critérios de inclusão supracitados.

A busca e seleção dos estudos se deu por dois revisores que realizaram independentemente a análise dos mesmos. Inicialmente, a partir da utilização dos DECS mencionados, juntamente com operadores Booleanos foram selecionados estudos publicados nos últimos 5 anos (2018–2023) seguido da análise de títulos e resumos. Nesta etapa, foram excluídos estudos com modelos animais, artigos de opinião, estudos que não consideravam a abordagem cirúrgica da EIA, bem como revisões de literatura.

Concluída tal etapa, procedeu-se a recuperação dos textos completos dos artigos para análise dos demais critérios de inclusão e exclusão. Foram excluídos ainda citações duplicadas e estudos não correspondentes aos parâmetros revisionais propostos. Possíveis discordâncias foram solucionadas com discussão com um terceiro revisor, sendo a inclusão decidida após consenso com os dois revisores principais.

A extração dos dados epidemiológicos e demográficos foi realizada com auxílio de planilha do Microsoft Excel (Microsoft Corp., Redmond, WA, EUA), incluindo parâmetros como número de pacientes, grau de curvatura inicial e final da EIA, estratégias de tratamento, recidivas, complicações, e resultados obtidos.

O protocolo da revisão sistemática foi registrado no *International prospective register of systematic reviews* (PROSPERO) sob o número ID CRD42023429455 visando melhor qualidade e adequação dos resultados aos objetivos propostos.

## Resultados

Após processo de triagem conforme protocolo PRISMA foram inicialmente recuperados 83 estudos, destes, 8 eram guias de prática clínica, 7 revisões sistemática e 21 eram estudos de opinião e/ou revisão narrativa. Também foram excluídos estudos fora da temática ( *n* = 13) e estudos de abordagem conservadora da EIA ( *n* = 25). Foram consideradas para análise 8 estudos as variáveis: técnicas utilizadas, redução de curvatura, melhora e/ou manutenção da função pulmonar, além da redução da dor e/ou melhora da qualidade de vida ( Fig. 1 ).

**Fig. 1:** Triagem e seleção de estudos conforme metodologia PRISMA. **Fonte:** Autoria própria (2023).

A amostra total destes estudos foi de 638 adolescentes com EIA, com uma idade média de 14,93 anos ± 1,24. A correção média da curvatura patológica principal nos estudos foi de 55,06% ± 12,24 ( Tabela 1 ). 15 16 17 18 19 20 21

**Tabela 1:** Correlação entre idade de intervenção cirúrgica e correção média da curvatura patológica principal em adolescentes com EIA

Estudo	Amostra	Idade média	Correção média da curvatura patológica
** Byun e col. 15**	35 pacientes com EIA torácica	14,9 anos	49,19%
** Grabala et al. 18**	195 adolescentes com EIA	14,3 anos	59%
** Zhang et al. 19**	11 adolescentes com AC > 130°	13,36 anos	40,39%
** Altaf et al. 25**	37 adolescentes com EIA	14,6 anos	33,4%
** Santos et al. 16**	41 adolescentes com EIA	17,8 anos	68%
** Sapriza et al. 17**	19 pacientes com EIA	14 anos	60%
** Garcia et al. 20**	278 pacientes com EIA operados com fixações seletiva, tradicional e múltipla.	15 anos	71%
** Dittmar-Johnson et al. 21**	22 pacientes submetidos à cirurgia corretiva para EIA	15,5 anos	59,5%

Abreviaturas: AC, ângulo de Cobb; EIA, escoliose idiopática do adolescente.

Fonte: Elaboração própria (2023).

A Fig. 2 representa a análise da correção média da curvatura patológica através dos ACs pré e pós-operatório. 15 16 17 18 19 20 21

**Fig. 2:** Correção média da curvatura patológica através do ângulo de Cobb pré e pós-operatório.

## Discussão

## Correção média das Curvaturas Patológicas

Os principais objetivos da abordagem cirúrgica da EIA são a correção coronal e sagital das curvaturas patológicas e evitar a progressão destas. Assim, espera-se que haja após o tratamento a correção da curva primária principal, e ainda correção de curvas secundárias com manutenção de uma lordose lombar e cifose torácica adequadas. É objetivo do tratamento a obtenção de um equilíbrio entre a fusão do menor número de seguimentos móveis possível e a redução/correção da deformidade existente. Associado à correção das deformidades são considerados resultados esperados ainda a melhora e/ou manutenção da função pulmonar, redução da dor e melhora da qualidade de vida. 14

Todos os estudos selecionados utilizaram técnicas de fusão espinhal posterior para correção da EIA. Uma das técnicas descritas é a fusão espinhal posterior com parafuso pedicular segmentar em uma lesão lombar e gancho e fiação segmentar em uma lesão torácica (construção híbrida) usando o sistema de instrumentação ISOLA (DePuy AcroMed, Raynham, MA, EUA) sem toracoplastia. 15 Foram ainda relatadas técnicas de rotação vertebral direta (RVD) associadas à osteotomia tipo 1, 16 Artrodese torácica, artrodese toracolombar e artrodese lombar. 17 22 23

## Função Pulmonar Após Intervenção Cirúrgica

Para correção da EIA podem ser utilizadas técnicas de fusão posterior ou anterior da coluna vertebral. Embora os dois tipos de abordagem ofereçam correção das curvaturas patológicas, alguns estudos sugerem que a abordagem anterior com toracotomia pode resultar em maior comprometimento da função pulmonar. A função pulmonar nos estudos foi avaliada conforme os valores de capacidade vital forçada (CVF) e volume expiratório forçado em 1 segundo (VEF1). 15 24

No estudo longitudinal com acompanhamento por 15 anos realizado por Byun et al., 15 dentre os pacientes que não apresentavam comprometimento da CVF no pré-operatório ( *n* = 35) observou-se que não foram identificadas diferenças estatisticamente significativas nas porcentagens médias pré-operatórias (74,0 ± 19,8%) e pós-operatórias (76,4 ± 16,0%), com *p* = 0,63. Por outro lado, os pacientes que já possuíam comprometimento pré-operatório se beneficiaram da abordagem cirúrgica. 15

Em abordagem similar, incluindo 39 pacientes com EIA e idade entre 10 e 21 anos observou-se que após a restauração da cifose torácica houve melhora do VEF1 de 2,74 para 2,98L ( *p* = 0,005) e CVF de 3,23 para 3,47L ( *p* = 0,008), contudo, a capacidade pulmonar total não se alterou após 5 anos de acompanhamento. 24

Em um dos estudos, incluindo 88 pacientes com EIA grave e 107 com EIA moderada e uma correção média da deformidade de 59%, foram relatados ganhos significativos na função pulmonar. Observou-se após 2 anos uma melhora na CVF de 69,9% e VEF1 de 81% no grupo de EIA grave. No grupo EIA moderada o VEF1 também melhorou significativamente (76,9%) após 2 anos de seguimento. 19

Em relação ao tipo de fixação vertebral um dos estudos discutiu diferentes possibilidades de fixação vertebral considerando o grau de correção de EIA necessário. O estudo refere-se ao tratamento de três grupos de pacientes com EIA submetidos a três diferentes abordagens de fixação: tradicional, seletiva e múltipla. No primeiro grupo operado, foi fixada toda a extensão das curvaturas estruturadas, conhecida como fixação tradicional; no grupo da fixação seletiva, apenas a curva principal foi fixada com a intenção de preservar a mobilidade vertebral. Já no terceiro grupo, a fixação múltipla ocorreu conforme o teste de flexibilidade, definindo o grau de correção necessário e vértebras a serem fixadas. No estudo, as técnicas tradicionais e fixação múltipla tiveram correção de 75% e 78%, respectivamente, da curvatura patológica, enquanto a correção da fixação seletiva foi de 60%. Considerando todos os casos, o estudo apresentou uma correção média de 71% ± 7,87 das curvaturas patológicas da EIA. O estudo não abordou qualidade de vida ou função pulmonar associados aos resultados obtidos. 20

Sapriza et al. 17 acompanharam 19 pacientes com EIA submetidos à correção cirúrgica por fusão espinhal posterior ao longo de 9,5 anos, com uma média de 10 espaços intervertebrais fundidos por paciente. O valor médio do ângulo pré-operatório foi de 58 (variando de 90–42). O valor médio do ângulo pós-operatório antes do final do 1° ano foi de 23, para uma taxa de correção de 60%. O valor médio do ângulo ao final do seguimento foi de 26. Portanto, uma correção média de 5% foi perdida ao final do seguimento. Apenas 3 casos tiveram perda superior a 10%. Os adolescentes consideraram significativa melhoria da qualidade de vida e aparência corporal após o período de seguimento. Os autores não analisaram repercussões pulmonares no estudo.

Santos et al. 16 realizaram um estudo observacional transversal com 43 adolescentes (idade média 17,8 anos) e AC entre 35 e 55° (AC médio de 51,5° ± 13,7°). Foi utilizada a abordagem posterior com acesso clássico pela linha média. Após dissecção subperiosteal da musculatura, foi realizada a osteotomia tipo 1 de Schwab. Ainda, o nível mais proximal de artrodese e osteotomias de Schwab tipo 2 foram realizadas nas vértebras periapicais de acordo com a avaliação subjetiva da redutibilidade da curvatura durante o procedimento. Obteve-se uma correção de 68% da curva torácica principal. Não foram relatados no estudo dados sobre qualidade de vida, dor e/ou função pulmonar.

Em um estudo realizado com 195 adolescentes com EIA, sendo 88 classificados como graves (AC > 130°) e 107 como EIA moderada, submetidos à abordagem posterior para correção cirúrgica da EIA, observou-se uma correção média da deformidade após abordagem cirúrgica de 59% e uma redução do número de indivíduos com queixas álgicas de 36 para 8%. Em pacientes com EIA grave ( *n* = 88), houve redução da curvatura média de 131 para 61° e da cifose torácica média de 83 para 35°. Já entre os pacientes com EIA moderada, a redução da curvatura média foi de 60° para 18°, não havendo redução da cifose torácica neste grupo. 18

Em estudo abordando a correção cirúrgica somente posterior com tração halo-femoral 11 adolescentes com AC > 130°, foram avaliados em um tempo médio de seguimento de 32,18 ± 8,17 meses. A média pré-operatória do AC coronal da curva maior foi de 139,01° ± 5,83°, e a flexibilidade média foi de 17,21% ± 3,33%. Após a abordagem obteve-se uma redução do AC coronal da curvatura maior para 82,98° ± 6,91° com taxa de correção de 40,39%. No seguimento final, a taxa de perda corretiva do AC foi de apenas 0,72%. 19

Já no estudo realizado com 37 pacientes com EIA moderado (AC entre 50 e 129°) submetidos à toracoplastia, observou-se uma curvatura pré-operatória média de 50,0° ± 12,4°, com posterior redução para 16,6° ± 6,3°. A correção média de deformidade foi de 33,4%, com uma melhora da CVF média de 55,4% e VEF1 de 72%. No estudo os autores utilizaram o escore *Scoliosis Research Society* (SRS) 22 para analisar a qualidade de vida dos adolescentes, sendo constatada uma melhora do escore de 3,8 para 4,3 após abordagem cirúrgica. 25

Em estudo realizado por Dittmar-Johnson et al., 21 foram incluídos 22 pacientes submetidos à fusão espinhal posterior para correção da EIA com curvas patológicas superiores à 45°. Os pacientes possuíam uma média etária de 15,5 anos. A aplicação do questionário SRS 22 gerou os seguintes escores médios: dor 4,6, função 4,3, autoimagem 4,41, saúde mental 4,89 e satisfação 5,0. Sendo assim, considerou-se que a intervenção cirúrgica permitiu boa qualidade de vida nos cinco parâmetros avaliados.

O SRS 22 também já havia sido utilizado anteriormente em longitudinal com 10 anos de acompanhamento de 109 adolescentes com EIA tratados com abordagem cirúrgica. Quando todos os pacientes foram incluídos, o AC médio pré-operatório das curvas maiores no plano frontal foi de 60,8° +/− 17,5°. Curvas maiores que foram corrigidas em 38,7° +/− 22,1% nas radiografias de flexão, no pós-operatório, alcançaram uma correção de 64,0° +/− 15,8%. Na última visita de acompanhamento, 10,3° +/− 10,8° de perda de correção foram registrados nas curvas principais no plano frontal com 50,5° +/− 23,1% de taxa de correção final. Além disso, os ângulos médios de cifose pós-operatória e final e os ângulos de lordose lombar foram de 37,7° +/− 7,4°, 37,0° +/− 8,4°, 37,5° +/− 8,7° e 36,3° +/− 8,5°, respectivamente. No geral, 4 pacientes (3,7%) tiveram falha do implante. Infecção superficial precoce foi observada em 3 (2,8%) pacientes. No geral, as pontuações médias do questionário SRS-22 para autoimagem geral, função, estado mental, dor e satisfação com o tratamento foram 3,8 +/− 0,7, 3,6 +/− 0,7, 4,0 +/− 0,8, 3,6 +/− 0,8, e 4. 6 +/− 0,3, respectivamente, na última visita de acompanhamento. Frente aos resultados, os autores consideraram que a abordagem cirúrgica é eficiente na correção das deformidades em planos frontal, sagital e equilíbrio do tronco, resultando em melhor qualidade de vida. 26

## Considerações Finais

Em todos os estudos selecionados com fusão espinhal posterior para correção da EIA, verificou-se redução significativa das curvaturas patológicas (> 49%), sendo que a reincidência de curvatura em nenhum dos estudos superou um ganho patológico superioraà 5%. Quanto à função pulmonar, os estudos apontaram ganhos significativos de VEF1 e CVF em pacientes com EIA grave, não foram relatados prejuízos de função pulmonar após intervenção cirúrgica para correção da EIA. Por fim, a maior parte dos estudos relatou ganhos em qualidade de vida e redução da dor após a intervenção cirúrgica.
